# Robotic and clinical evaluation of upper limb motor performance in patients with Friedreich’s Ataxia: an observational study

**DOI:** 10.1186/s12984-015-0032-6

**Published:** 2015-04-23

**Authors:** Marco Germanotta, Gessica Vasco, Maurizio Petrarca, Stefano Rossi, Sacha Carniel, Enrico Bertini, Paolo Cappa, Enrico Castelli

**Affiliations:** Don Carlo Gnocchi Onlus Foundation, Piazzale Morandi 6, 20121 Milan, Italy; Movement Analysis and Robotics Laboratory (MARLab), Neurorehabilitation Units, IRCCS Bambino Gesù Children’s Hospital, Via Torre di Palidoro, 00050 Passoscuro (Fiumicino) Rome, Italy; Unit of Neuromuscular and Neurodegenerative Disorders, Laboratory of Molecular Medicine, IRCCS Bambino Gesù Children’s Hospital, Piazza S. Onofrio 4, 00165 Rome, Italy; Department of Economics and Management - Industrial Engineering (DEIM), University of Tuscia, Via del Paradiso 47, 01100 Viterbo, Italy; Department of Mechanical and Aerospace Engineering, “Sapienza”, University of Rome, Via Eudossiana 18, 00184 Roma, Italy

**Keywords:** Friedreich’s ataxia, Robot-mediated evaluation, Scale for the Assessment and Rating of Ataxia, Upper limb, Reaching task, Kinematics, Accuracy, Smoothness, Submovements

## Abstract

**Background:**

Friedreich’s ataxia (FRDA) is the most common hereditary autosomal recessive form of ataxia. In this disease there is early manifestation of gait ataxia, and dysmetria of the arms and legs which causes impairment in daily activities that require fine manual dexterity. To date there is no cure for this disease. Some novel therapeutic approaches are ongoing in different steps of clinical trial. Development of sensitive outcome measures is crucial to prove therapeutic effectiveness. The aim of the study was to assess the reliability and sensitivity of quantitative and objective assessment of upper limb performance computed by means of the robotic device and to evaluate the correlation with clinical and functional markers of the disease severity.

**Methods:**

Here we assess upper limb performances by means of the InMotion Arm Robot, a robot designed for clinical neurological applications, in a cohort of 14 children and young adults affected by FRDA, matched for age and gender with 18 healthy subjects. We focused on the analysis of kinematics, accuracy, smoothness, and submovements of the upper limb while reaching movements were performed. The robotic evaluation of upper limb performance consisted of planar reaching movements performed with the robotic system. The motors of the robot were turned off, so that the device worked as a measurement tool. The status of the disease was scored using the Scale for the Assessment and Rating of Ataxia (SARA). Relationships between robotic indices and a range of clinical and disease characteristics were examined.

**Results:**

All our robotic indices were significantly different between the two cohorts except for two, and were highly and reliably discriminative between healthy and subjects with FRDA. In particular, subjects with FRDA exhibited slower movements as well as loss of accuracy and smoothness, which are typical of the disease. *Duration of Movement*, *Normalized Jerk*, and *Number of Submovements* were the best discriminative indices, as they were directly and easily measurable and correlated with the status of the disease, as measured by SARA.

**Conclusions:**

Our results suggest that outcome measures obtained by means of robotic devices can improve the sensitivity of clinical evaluations of patients’ dexterity and can accurately and efficiently quantify changes over time in clinical trials, particularly when functional scales appear to be no longer sensitive.

## Background

Friedreich’s ataxia (FRDA) is the most common hereditary autosomal recessive form of ataxia resulting from the homozygous expansion of a guanine–adenine–adenine (GAA) trinucleotide repeat in intron 1 of the frataxin gene on chromosome 9q13. FRDA affects about 1 in 30,000 individuals in Western Europe [1]. The clinical features of FRDA are progressive ataxia, weakness, spasticity, sensory symptoms and cardiomyopathy [2,3].

In patients with FRDA, gait ataxia and general clumsiness are the commonest presenting symptoms and upper limb ataxia progresses slower than lower limb impairment, thus the majority of non-ambulatory patients can still use their upper limbs for daily activities. Consequently, specific tools for assessing upper limb function may then be suitable both for ambulatory and non-ambulatory patients and valuable for long-term evaluation [4].

Currently, the effectiveness of treatment is generally measured by clinical scales that include several functional tests like the Friedreich Ataxia Rating Scale (FARS) [5], the International Cooperative Ataxia Rating Scale (ICARS) [6], the brief version of ICARS named Brief Ataxia Rating Scale (BARS) [7], and the Scale for the Assessment and Rating of Ataxia (SARA) [8]. SARA was recognized as the most sensitive scale for its high construct validity, best effect size and for its compact nature in a longitudinal analysis of a numerous cohort of 96 FRDA patients in comparison with ICARS and FARS [9,10], although the scores of these scales are well correlated with each other. The applicability of these functional scales in children is still an open question, as age-validation is needed. However, SARA was demonstrated to be suitable with a good reliability in healthy children beyond the age of 10. Moreover, in a preliminary pilot study concerning age-dependency, it emerges that SARA is more suitable for long-term quantitative ataxia assessment from child to adulthood in comparison to ICARS and BARS [11].

In general, however, clinical evaluations exhibit several limitations: a low rate of reproducibility, low resolution, lack of sensitivity, and floor and ceiling effects [12]. Therefore, in the last few years, researchers have developed a growing interest in the quantitative evaluation techniques of residual motor abilities, especially those focusing on the upper limb function. Such efforts have been primarily motivated by the inherent ability of robotic devices to objectively quantify motor performance and to detect small variations; consequently, robot mediated evaluations could represent a useful additional tool for clinical measures [13,14]. Briefly, robot devices are effectively employed to assess motor recovery of the upper limbs mainly in patients with stroke [15-22] (for reviews, see [23] and [24]) and in children with cerebral palsy [25-28] and the mounting evidence suggests that robotic outcomes can also be effectively employed in other diseases, such as Multiple Sclerosis [29].

Up to now, only a few studies have aimed to a quantitative evaluation of the motor performance of upper limbs in patients with ataxia [30-34] and even fewer studies focused on individuals with FRDA. Day *et al.* [35] reported that analyzing the influence of vision on upper limb reaching movements by using an optical tracker system, FRDA patients showed prolonged reaction times and less accurate and slower movements compared to healthy subjects. Bardorfer *et al.* [36], by using an haptic interface, showed that FRDA patients were able to perform tracking tasks, but with lower velocity and less accuracy than a healthy control group; the relationship between the robotic indices and clinical scales was not investigated and the movement was not partitioned into submovements, which were recognized as significant for the analysis of neurological disease progression [37,38]. Finally, Maurel *et al.* [39] developed and applied an upper limb kinematic protocol adapted to children and young adults with FRDA; they highlighted lower values of velocity, precision and smoothness of movements of upper limbs in three tasks - i.e. pointing, circle-drawing and prono-supination tasks - in comparison with a control group.

Therefore, the aim of this study was to assess with a robotic system the upper limb performances in a cohort of children and young adults affected by FRDA and age and gender-matched with healthy subjects. We analyzed the kinematics, accuracy, smoothness, and submovements of the upper limb during a planar point-to-point reaching task in a dual-modal visual-haptic feedback by means of the InMotion Arm Robot, the commercial version of the MIT-Manus [40].

Specifically, the purposes of this paper are threefold. Firstly, to study the discriminative sensitivity of the selected indices computed via the robotic system between healthy subjects and patients with FRDA. Secondly, to assess the reliability of the selected indices. Finally, to evaluate the correlation between the indices provided by the robotic system and a range of clinical and disease characteristics in subjects with FRDA. We hypothesized that the indices of upper limb performance would be associated with disease severity, as evaluated by SARA, and with disease-related variables, as disease duration and number of GAA repeats, which are variables well known in literature highly related with the phenotype [41].

## Methods

### Subjects

Fourteen genetically confirmed individuals affected by FRDA (mean age 15.3 years, range 6–28 years, 4 males, 10 females) were recruited at the Neurorehabilitation Division of the IRCCS Bambino Gesù Children’s Hospital (Rome, Italy). Eleven patients were able to walk without assistance, two patients needed walking aids, and one was non ambulatory. Eye movements were evaluated in all patients as a part of the neurological evaluation. Almost all patients had mild oculomotor abnormalities with fixation instability, square wave jerks, and, rarely, nystagmus. None of them wore lens for visual refractive deficit or presented limitation of Range of Motion at the level of the elbow and the shoulder. Clinical features and genetic information for FRDA patients, together with demographic data, are reported in Table 1. The status of the disease was scored using SARA. Eighteen age and gender-matched [42,43] healthy subjects (mean age 15.1 years, range 7–28 years, 5 males, 13 females) were also enrolled as a control group. Inclusion criteria for healthy subjects were absence of neurological and visual deficits, and a physiological Range of Motion for elbow and shoulder.

**Table 1:** **Demographic data and clinical information for patients with Friedreich’s ataxia.**

**FRDA subjects**	**Sex**	**Hand dominance**	**Age (years, months)**	**SARA (total score)**	**SARA (upper limb score)**	**GAA smaller**	**GAA longer**	**Disease duration (years)**
1	F	R	9, 3	9.5	3	780	1115	3
2	F	R	12, 7	21.5	4	633	633	6
3	F	R	14, 4	15.5	7.5	930	1066	7
4	F	R	6, 1	3.5	1.5	682	848	1
5	M	R	13, 1	14	3	600	750	6
6	F	R	28, 1	6	1	na	na	8
7	F	L	14, 9	9	2	715	900	7
8	M	R	14, 1	10	3	780	780	6
9	M	R	28, 0	2.5	0.5	na	na	9
10	F	R	8, 6	9	3	1014	1347	3
11	F	R	14, 9	8	2	264	1347	5
12	F	R	17, 1	24.5	6,5	900	1000	8
13	M	R	14, 3	9.5	2,5	805	1064	5
14	F	R	26, 7	39	16	790	790	12

na: not available.

All the subjects, except for one FRDA patient, were right handed. Hand dominance was established as the hand that participants used for writing and personal activities. All subjects were naïve to the robotic device and the task.

The Ethics Committee of the Children’s Hospital approved the experimental protocol, which was explained, together with the aims of the research, to the subjects involved in the study and children’s parents. Written consent was obtained from all adults and children’s parents.

### Equipment

**Figure 1:**
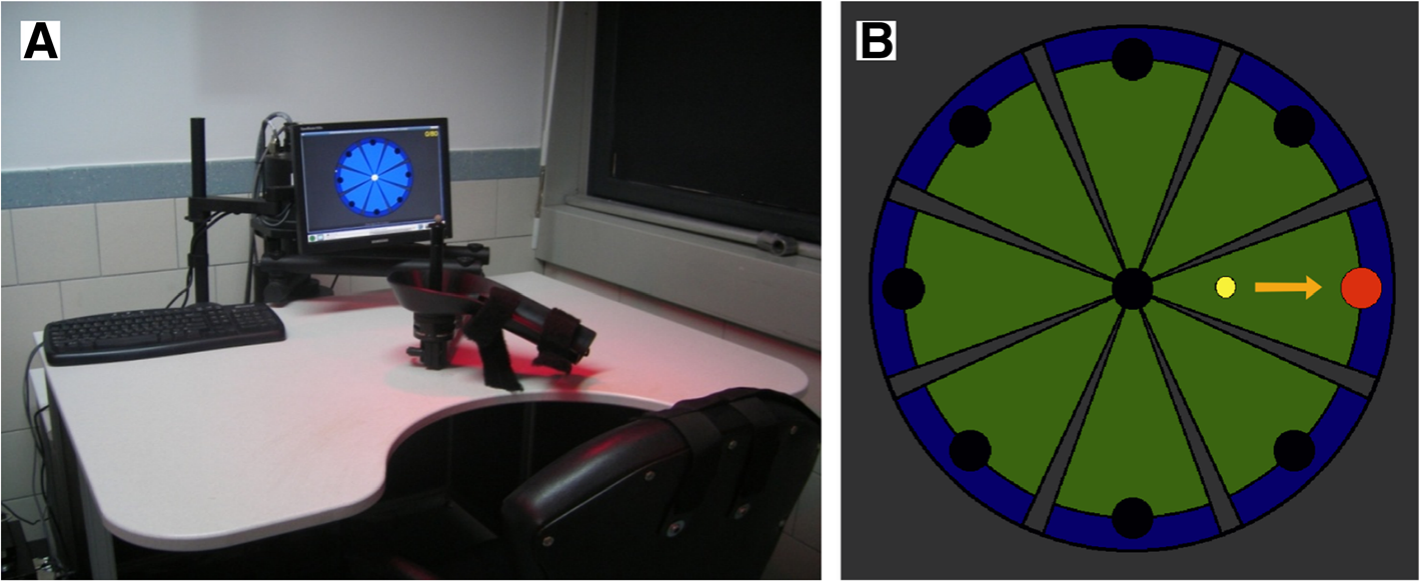
**(A)** Robotic system for upper limb rehabilitation; **(B)** Visual template of the reaching task. The yellow circle indicates the position of the end-effector while the target to be reached is showed by the red circle.

The assessment of upper limb motor performance was conducted by means of the InMotion Arm Robot (Interactive Motion Technologies Inc., Watertown, MA - USA, see Figure 1A), a robot designed for clinical neurological applications [44]. It is based on a direct-drive, five-bar-linkage SCARA (Selective Compliance Assembly Robot Arm) mechanism that provides two translational degrees of freedom, restricting the hand motion to the horizontal plane. When motors are turned off, the highly backdrivable, low-friction robot does not interfere with motion and allows the individual to freely move the end-effector. It is also equipped with sensors that provide the position of the end-effector (with an accuracy of 100 μm) with a sampling rate of 200 Hz. A screen located in front of the subject shows the position of the end-effector, together with the exercise to be performed.

### Clinical assessment

The status of the disease was rated by the same neurologist specializing in ataxia by using the Scale for the Assessment and Rating of Ataxia (SARA) [8], a clinical scale with total scores ranging from 0 (no ataxia) to 40 (most severe ataxia). It includes eight items: (i) gait (0–6 points); (ii) stance (0–6 points); (iii) sitting (0–4 points); (iv) speech disturbance (0–6 points); (v) finger chase (0–4 points); (vi) nose-finger test (0–4 points); (vii) fast alternating hand movement (0–4 points); and, finally, (viii) heel-shin slide (0–4 points). Upper limb impairment score of SARA is the sum of the previously indicated items v, vi and vii.

### Robotic evaluation

The robotic evaluation of upper limb performance consisted of planar reaching movements performed with the robotic system. Subjects were comfortably seated on a chair, with their hand grasping the end-effector. In order to minimize compensatory movements, the trunk was restrained by a five-point seatbelt. An orthotic device supported the forearm and the hand to prevent all wrist movements and forearm prono-supination. The center of the workspace was located in front of the subject at the midline of the body. The position (height and distance from the table) of the chair was set depending on the subject’s anthropometric measures, so that, handling the end-effector with the orthotic device in the center of the workspace, the elevation angle of the shoulder was 45 degrees for all the participants.

The protocol consisted of five blocks of 16 unassisted planar reaching movements, making a total of 80 reaching movements. In a single block, eight white targets equally spaced on a circumference (with radius of 14 cm), and a white target positioned in the center of the circumference were shown on the screen located in front of the subject, together with the end-effector position (Figure 1B). The center of the circumference was coincident with the center of the workspace. Starting from the center, subjects were asked to move the end-effector, with a self-selected speed, in order to reach the blinking target and to come back to the center following the visual feedback in a virtual environment, along a straight path of about 14 cm; additionally the participants were not asked to perform the task with a specific time constraint and, then, the movement accuracy was implicitly a task requisite. The trial involves only the shoulder and elbow planar coordination. The sequence of center-out movements was randomized. The motors of the robot were turned off, so that the device worked as a measurement tool. Only the dominant arm was tested. The session per subject/patient lasted less than 20 minutes. Both patients and healthy subjects were tested twice, to assess the test-retest reliability of the proposed outcome measures. The time interval between testing was 1–7 days.

### Data analysis

Data measured by the robot were processed offline to obtain quantitative indices related to different features of the subject’s dexterity. The recorded end-effector position was filtered with a 6^th^ order zero phase shift low-pass Butterworth filter, with a cut-off frequency of 10 Hz, and differentiated to obtain speed, acceleration, and jerk. Then, from the global measure, we identified the 80 reaching movements: specifically, each movement was considered to start when the speed magnitude became greater than 10% of the peak speed and the movement was considered to end when the speed dropped and remained below the 10% of the peak speed [20]. For each movement, a set of indices was computed. Among feasible measures proposed in the literature on neuro-rehabilitation of the upper limb [20,45] to characterize movement smoothness, movement accuracy, and tracking rapidity, we selected the following indices grouped as: kinematic, accuracy, smoothness, and submovement indices.

### Kinematic indices

To characterize the kinematics of the movement, we measured: (i) the *Duration of Movement* (*D*), defined as the time between the movement onset and the movement termination, (ii) the *Mean Velocity* (*MV*) and (iii) the *Peak Velocity* (*PV*) values of the velocity profile [19].

### Accuracy indices

The *Length Ratio* (*LR*) is defined as the ratio between the path actually travelled by the subject and the desired one (*L*_*t*_), i.e. the minimum distance between the beginning and the end of the trajectory [20]:

1 $$ LR=\frac{{\displaystyle \sum dR}}{L_t} $$

where *dR* is the distance between two points of the trajectory. Higher values of *LR* represent a lower accuracy value.

The Lateral Deviation (*LD*) is defined as the highest deviation from the straight line that connects the initial and the final target position in the analyzed movement [27]. The *LD* value increases when accuracy decreases.

The Aiming Angle (*AA*) is computed as the angle between the line connecting the starting and ending target, and the line from the starting point to the peak speed point [19]. An *AA* decrease corresponds to an increase of accuracy.

### Smoothness indices

The Normalized Jerk (*NJ*) is expressed by the following equation [46]:

2 $$ NJ=\sqrt{\frac{1}{2}{\displaystyle \int {\mathbf{j}}^2\cdot \frac{T^5}{{\left({\displaystyle \sum dR}\right)}^2}\cdot dt}} $$

where **j** is the jerk, i.e. the derivative of acceleration, and *T* is the duration of the movement. Lower values of *NJ* indicate smoother movements.

The Speed Metric (*SM*) is measured as the ratio between the mean and the peak speed [13]:

3 $$ SM=\frac{v_{mean}}{v_{peak}} $$

The *SM* value increases when smoothness increases.

### Submovement indices

With respect to the submovements, we followed the approach proposed by Friedman *et al.* [47]. Specifically, we decomposed the reaching movements into submovements, modeled according to minimum jerk criterion with a bell-shape velocity profile [48]:

4 $$ \dot{x}(t)=\frac{A}{D}\left[30{\left(\frac{t-{t}_0}{D}\right)}^4-60{\left(\frac{t-{t}_0}{D}\right)}^3+30{\left(\frac{t-{t}_0}{D}\right)}^2\right] $$

where *D*, *A* and *t*_*0*_ are the duration, the amplitude and the starting time of a single submovement, respectively. Each velocity profile *F*(*t*) of the reconstructed movement is then composed of the overlap of *N* submovements:

5 $$ F(t)={\displaystyle \sum_{i=1}^N\left\{\begin{array}{ll}0\hfill & t<{T}_{0i}\hfill \\ {}\dot{x}(t)\hfill & {T}_{0i}\le t\le {T}_{0i}+D\hfill \\ {}0\hfill & {T}_{0i}+D\hfill \end{array}\right.} $$

Since the robot restrained the movement to the horizontal plane, each movement was implicitly two-dimensional, so it was defined by four parameters: duration *D*, starting time *T*, and amplitude in x and y direction (*A*_*x*_ and *A*_*y*_). Submovements were extracted from the measured velocity profile by using the constrained nonlinear optimization function (*fmincon*) in the Optimization toolkit of Matlab (MathWorks, Natick, MA - USA). For a given number of submovements, all the parameters were optimized simultaneously by minimizing the reconstruction error:

6 $$ E={\displaystyle \sum \frac{{\left[{F}_x(t)-{G}_x(t)\right]}^2+{\left[{F}_y(t)-{G}_y(t)\right]}^2+{\left[{F}_v(t)-\sqrt{G_x{(t)}^2+{G}_y{(t)}^2}\right]}^2}{2\left[{G}_x{(t)}^2+{G}_y{(t)}^2\right]}} $$

where *G*_*x*_(*t*) and *G*_*y*_(*t*) are the components of the measured end-effector velocity, *F*_*x*_(*t*) and *F*_*y*_(*t*) are the reconstructed *x* and *y* components of the velocity, and *F*_*v*_ is the reconstructed tangential velocity. Referring to the constraints, submovements were allowed to have a duration of at least 167 ms, following Rohrer et Hogan [49]; *A*_*x*_ and *A*_*y*_, instead, were limited to the size of the workspace, i.e., between −0.2 m and 0.2 m [50]. The optimization was run for an increasing number of submovements, until the error *E* is lower than a threshold, set to 0.02 [38].

Starting from the obtained submovements, the following indices were then computed: *Number of Submovements* (*NS*), *Duration of Submovements* (*DS*) and *Amplitude of Submovements* (*AS*) [38].

### Statistical analysis

All data were tested for normality with the Shapiro-Wilk test. When the assumption of normality was met, we assessed the differences between FRDA patients and the control group by using an independent t-test, with Welch’s correction when the variances were not equal. Otherwise, we used a Mann-Withney U test.

Reliability of parameters was analyzed using the Intraclass Correlation Coefficient (ICC) with an ICC(2,k) model. Reliability was classified as excellent (ICC ≥ 0.90), very good (ICC ≥ 0.80), good (ICC ≥ 0.70), moderate (ICC ≥ 0.6) or poor otherwise.

Finally, within-subject relations between robotic indices and clinical parameters (disease duration, the smaller GAA repeat size, the larger GAA repeat size, and the SARA scale) were tested with a Spearman’s rank order correlation, with a False Discovery Rate correction for multiple comparison. The significance level was set at 0.05 for all statistical tests.

Statistical analysis was performed with built-in functions of SPSS 21 (IBM, Armonk, NY - USA).

## Results

### Robotic indices

**Figure 2:**
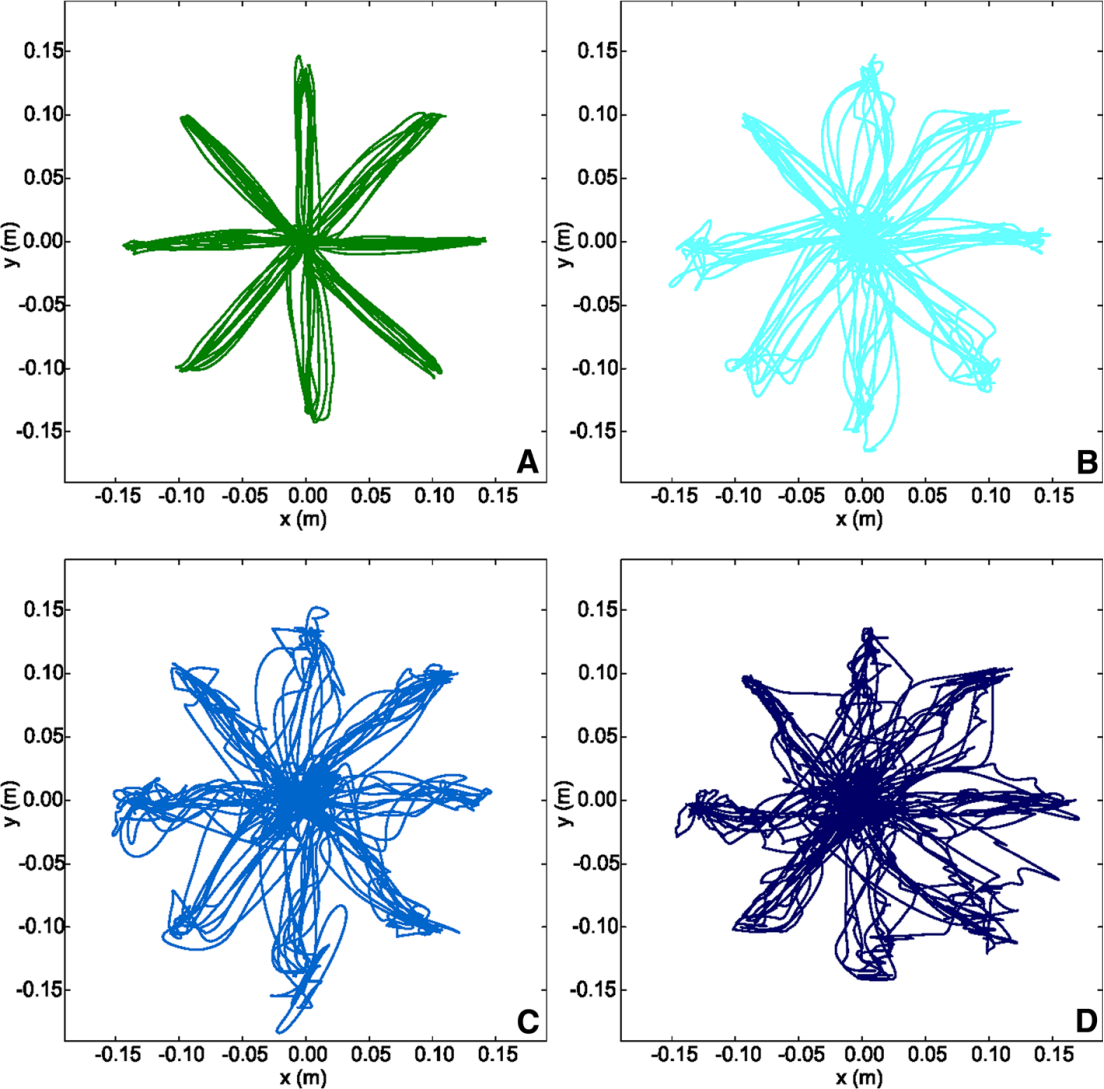
Plot of the path traced by a representative subject from the control group **(A)**, and by three FRDA patients with different SARA scores: 15 **(B)**, 24.5 **(C)**, and 39 **(D)**.

Figure 2 show the end-effector trajectories performed by a representative healthy subject and three patients with FRDA.

**Figure 3:**
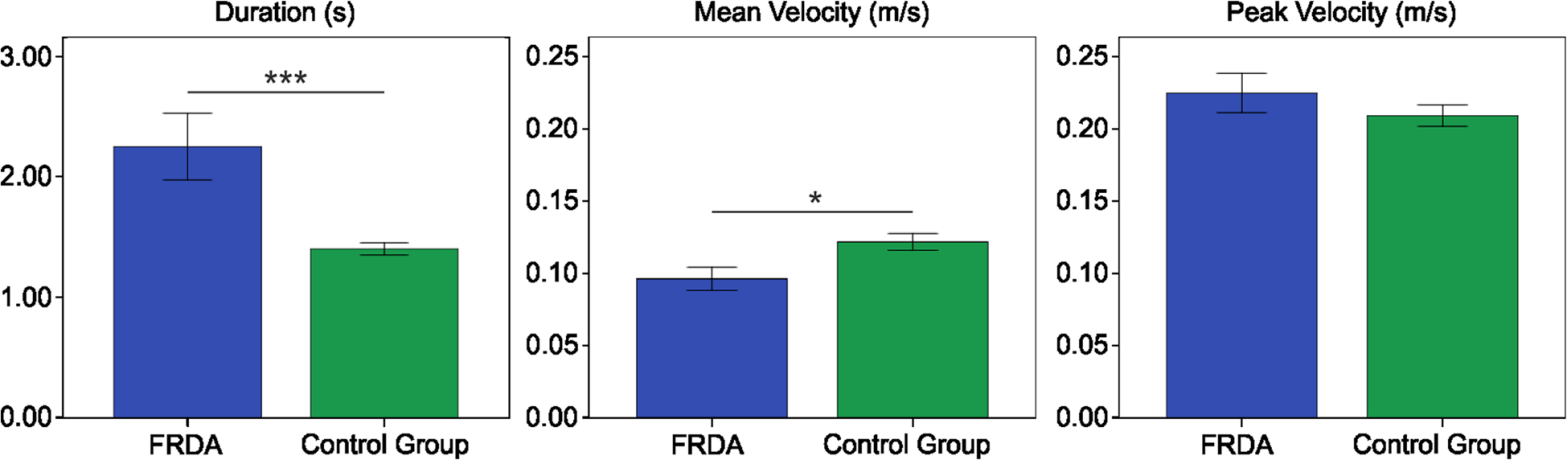
Kinematic indices. Means of the Duration of Movement (left), Mean Velocity (middle), and Peak Velocity (right), with error bars representing standard error. The symbol * indicates a significant difference between the two groups (p < 0.05); the symbol *** indicates a significant difference between the two groups (p < 0.001).

Figure 3 reports on the results of the kinematic indices. FRDA patients performed the required task with higher *D* values than the control group (FRDA: 2.25 ± 1.04 s; control group: 1.40 ± 0.21 s; U = 247, p < 0.001). Referring to the velocity profiles, the *MV* values were statistically different between the two groups (FRDA: 0.10 ± 0.02 m/s; control group: 0.12 ± 0.02 m/s; t = 2.644, p < 0.05); the *PV* values, instead, were not statistically meaningful (FRDA: 0.22 ± 0.05 m/s; control group: 0.21 ± 0.03 m/s; t = 1.176, p = 0.249).

**Figure 4:**
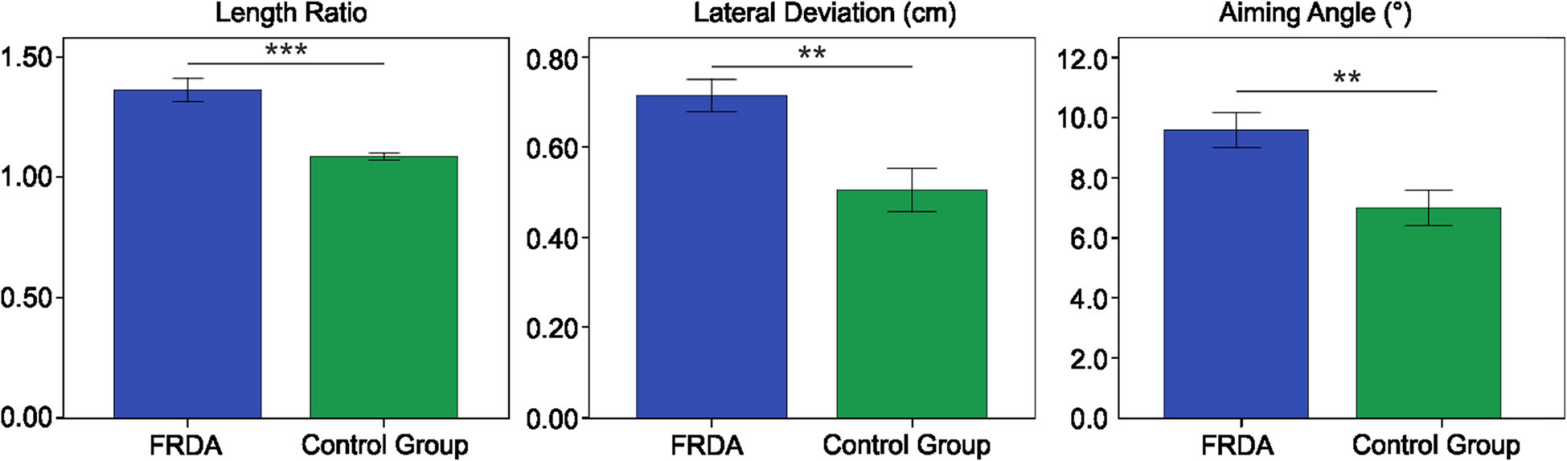
Movement accuracy indices. Means of the Length Ratio (left), Lateral Deviation (middle), and Aiming Angle (right), with error bars representing standard error. The symbol ** indicates a significant difference between the two groups (p < 0.01); the symbol *** indicates a significant difference between the two groups (p < 0.001).

Regarding the movement accuracy (Figure 4), all the selected indices showed a lower accuracy in the FRDA, compared with the control group; in fact, *LR* was higher in the FRDA patients than in the control group (FRDA: 1.37 ± 0.18; control group: 1.09 ± 0.06; U = 258, p < 0.001); *LD* was higher in the FRDA patients than in the control group (FRDA: 0.72 ± 0.13 cm; control group: 0.50 ± 0.20 cm; U = 213, p < 0.01); and, finally, the *AA* was higher in the FRDA patients than in the control group (FRDA: 9.59 ± 2.20°; control group: 6.85 ± 2.49°; t = 3.280, p <0.01).

**Figure 5:**
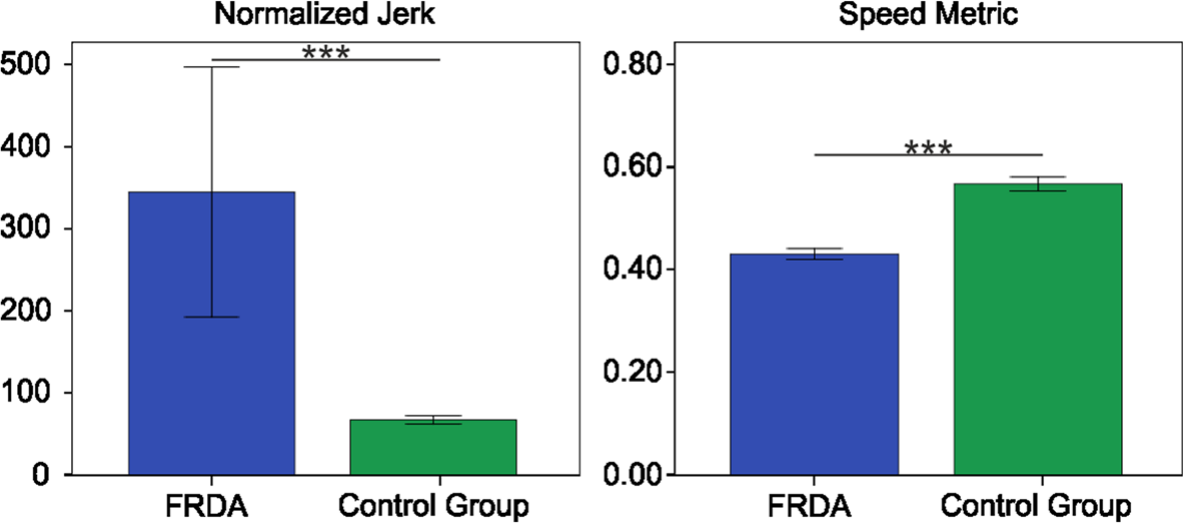
Movement smoothness indices. Means of the Normalized Jerk (left) and the Speed Metric (right), with error bars representing standard error. The symbol *** indicates a significant difference between the two groups (p < 0.001).

With respect to the movement smoothness (Figure 5), all the selected indices showed a lower smoothness in the FRDA, compared with the control group; in fact, *NJ* was higher in the FRDA patients than in the control group (FRDA: 333.24 ± 551.73; control group: 63.73 ± 20.95; U = 256, p < 0.001); *SM* was lower in the FRDA patients than in the control group (FRDA: 0.43 ± 0.04; control group: 0.57 ± 0.06; U = 5, p < 0.001).

**Figure 6:**
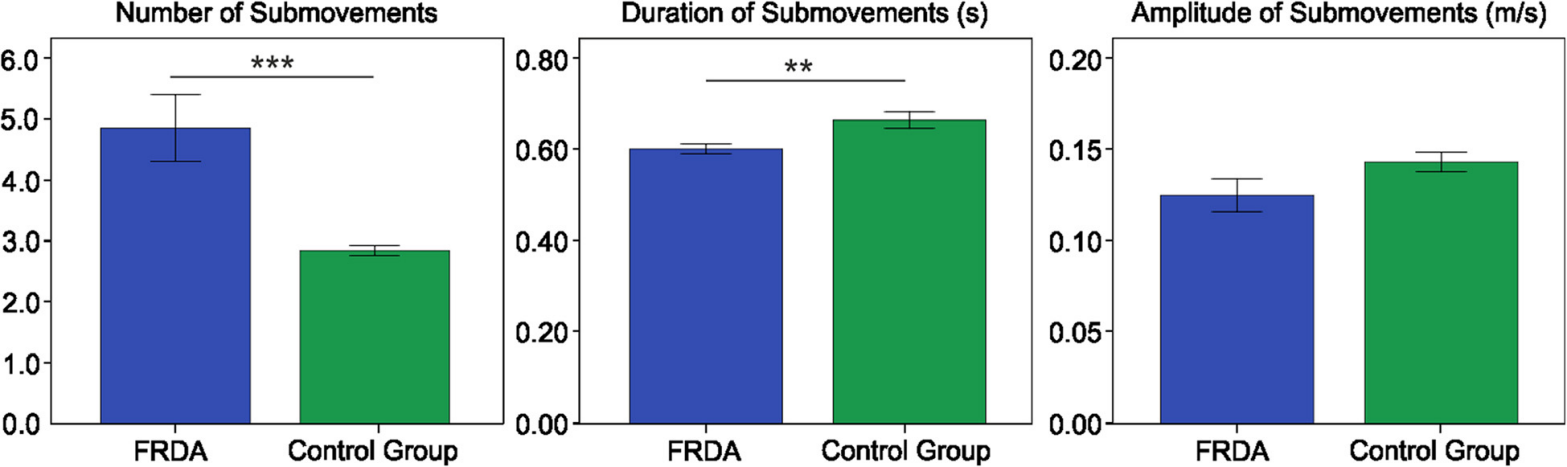
Submovement indices. Means of the Number of Submovements (left), Duration of Submovements (middle) and Amplitude of Submovements (right) with error bars representing standard error. The symbol ** indicates a significant difference between the two groups (p < 0.01); the symbol *** indicates a significant difference between the two groups (p < 0.001).

The results of the analysis of the submovements (Figure 6) revealed that movements in FRDA patients are made of a higher *NS* value compared to healthy subjects (FRDA: 4.84 ± 2.04; control group: 2.83 ± 0.34; U = 250, p < 0.001); moreover, the mean *DS* is lower in FRDA compared with control group (FRDA: 0.60 ± 0.04 s; control group: 0.67 ± 0.07 s; t = 3.022, p < 0.01). Finally, no differences were found between the two groups in *AS* (FRDA: 0.12 ± 0.03 m/s; control group: 0.14 ± 0.02 m/s; t = 1.820, p = 0.079).

### Test-retest reliability

The ICC values for the selected robotic indices both in FRDA patients and the control group are reported in Table 2. With respect to the FRDA group, the ICC values ranged from 0.686 for the *PV* (good reliability) to 0.969 for the *NS* (excellent reliability). With respect to the healthy subjects, all the selected indices showed a very good to excellent reliability: the ICC values ranged from 0.859 for the *LR* to 0.992 for the *LD*.

**Table 2:** **Robotic indices: values of Intraclass Correlation Coefficient (ICC) for patients with Friedreich’s ataxia and control group**

		**FRDA**	**Control group**
**Kinematic indices**	Duration of Movement	0.918	0.929
	Mean Velocity	0.785	0.910
	Peak Velocity	0.689	0.895
**Accuracy indices**	Length Ratio	0.802	0.859
	Lateral Deviation	0.845	0.992
	Aiming Angle	0.818	0.892
**Smoothness indices**	Normalized Jerk	0.879	0.914
	Speed Metric	0.686	0.901
**Submovement indices**	Number of Submovements	0.969	0.880
	Duration of Submovements	0.821	0.881
	Amplitude of Submovements	0.831	0.912

### Correlation analysis

The correlation analysis between robotic indices and clinical measures is reported in Table 3. The SARA scale and the upper limb score of the SARA subscale correlated moderately with the *D*, *NJ*, and *NS*. No correlation was found between the robotic indices and the smaller GAA repeat size or the larger GAA repeat size. Table 4 shows the correlation analysis for patients older than 10. From an analysis of this table and a comparative exam with Table 3, higher values of the Spearman’s correlation coefficient were found for the *D*, *NJ*, and NS indices, if compared with the ones found for the entire enrolled cohort. Moreover, moderate to high values of correlation were found (see Table 4) between the SARA scale, and the upper limb score of the SARA scale, with *MV*, *PV*, and *AS*.

**Table 3:** **Correlation between robotic indices and clinical parameters**

		**SARA (total score)**	**SARA (upper limb)**	**GAA smaller**	**GAA longer**	**Disease duration**
**Kinematic indices**	Duration of Movement	0.617*	0.659*	0.235	−0.361	−0.109
	Mean Velocity	−0.542	−0.634	−0.417	0.193	0.078
	Peak Velocity	−0.480	−0.541	−0.200	0.207	0.093
**Accuracy indices**	Length Ratio	0.355	0.363	−0.214	−0.336	−0.208
	Lateral	0.134	0.136	0.077	−0.102	−0.399
	Aiming Angle	0.029	0.160	0.238	0.224	−0.639
**Smoothness indices**	Normalized Jerk	0.674*	0.739*	0.291	−0.329	−0.118
	Speed Metric	−0.403	−0.385	−0.060	0.413	−0.100
**Submovement indices**	Number of Submovements	0.606*	0.708*	0.200	−0.266	−0.191
	Duration of Submovements	0.062	−0.009	0.109	−0.025	−0.051
	Amplitude of Submovements	−0.491	−0.605	−0.305	0.144	0.062

Spearman’s rank correlation coefficient are reported. The symbol * indicates a statistical significance, with p < 0.05 (p values are corrected for multiple comparison, by using a False Discovery Rate procedure).

**Table 4:** **Correlation between robotic indices and clinical parameters for patient older than 10 years**

		**SARA (total score)**	**SARA (upper limb)**	**GAA smaller**	**GAA longer**	**Disease duration**
**Kinematics indices**	Duration of Movement	0.891**	0.776**	0.117	−0.683	0.106
	Mean Velocity	−0.818**	−0.808**	−0.500	0.150	−0.115
	Peak Velocity	−0.727*	−0.690*	−0.233	0.117	−0.023
**Accuracy indices**	Length Ratio	0.564	−0.433	−0.433	−0.600	0.065
	Lateral Deviation	0.318	0.242	−0.017	−0.517	−0.185
	Aiming Angle	0.200	0.205	0.117	−0.217	−0.411
**Smoothness indices**	Normalized Jerk	0.964**	0.904**	0.233	−0.517	0.046
	Speed Metric	−0.500	0.217	0.217	0.850	−0.259
**Submovement indices**	Number of Submovements	0.927**	0.872**	0.050	−0.567	0.018
	Duration of Submovements	0.770	0.014	0.267	0.067	−0.129
	Amplitude of Submovements	−0.773*	−0.772*	−0.400	0.117	−0.148

Spearman’s rank correlation coefficient are reported. The symbol * indicates a statistical significance, with p < 0.05; the symbol ** indicates a statistical significance, with p < 0.01 (p values are corrected for multiple comparison, by using a False Discovery Rate procedure).

Finally, the correlation among the robotic indices for patients with FRDA is reported in Table 5. High correlation was found between *D*, *MV*, *PV*, *LR*, *NJ*, *NS* and *AS*. No correlation or lower correlation was found between *LD*, *AA*, *SM*, *DS* and the remaining above-mentioned indices. *DS* showed no correlation with all the other indices. *D*, *MV*, *NJ*, and *NS*, which show correlation with the SARA scale, are the indices that showed the highest correlation between them.

**Table 5:** **Correlation coefficients among the robotic indices for patients with FRDA**

	**Duration of movement**	**Mean velocity**	**Peak velocity**	**Length ratio**	**Lateral deviation**	**Aiming angle**	**Normalized jerk**	**Speed metric**	**Number of submovements**	**Duration of submovements**	**Amplitude of submovements**
**Duration of movement**	1	−0.793**	−0.741**	0.771**	0.323	0.200	0.960**	−0.604*	0.947**	0.099	−0.758**
**Mean velocity**	−0.793**	1	0.965**	−0.327	0.033	0.029	−0.837**	0.182	−0.802**	−0.182	0.978**
**Peak velocity**	−0.741**	0.965**	1	−0.297	0.112	0.187	−0.789**	0.108	−0.754**	−0.138	0.969**
**Length ratio**	0.771**	−0.327	−0.297	1	0.644*	0.376	0.714*	−0.802**	0.754**	−0.231	−0.310
**Lateral deviation**	0.323	0.033	0.112	0.644*	1	0.697*	0.367	−0.618*	0.358	−0.385	0.099
**Aiming angle**	0.200	0.029	0.187	0.376	0.697*	1	0.226	−0.248	0.270	0.007	0.086
**Normalized jerk**	0.960**	−0.837**	−0.789**	0.714*	0.367	0.226	1	−0.547	0.974**	−0.059	−0.820**
**Speed metric**	−0.604*	0.182	0.108	−0.802**	−0.618*	−0.248	−0.547	1	−0.556	0.305	0.125
**Number of submovements**	0.947**	−0.802**	−0.754**	0.754**	0.358	0.270	0.974**	−0.556	1	−0.077	−0.807**
**Duration of submovements**	0.099	−0.182	−0.138	−0.231	−0.385	0.007	−0.059	0.305	−0.077	1	−0.103
**Amplitude of submovements**	−0.758**	0.978**	0.969**	−0.310	0.099	0.086	−0.820**	0.125	−0.807**	−0.103	1

Spearman’s rank correlation coefficient are reported. The symbol * indicates a statistical significance, with p < 0.05; the symbol ** indicates a statistical significance, with p < 0.01 (p values are corrected for multiple comparison, by using a False Discovery Rate procedure).

## Discussion

In this work, we quantitatively evaluated the upper limb motor performance in a cohort of individuals affected by FRDA compared with an age and gender matched control group of healthy subjects, by using a rehabilitation robotic device. Specifically, we analyzed the dexterity in performing a planar point-to-point reaching task, a multijoint movement that requires the coordination of shoulder and elbow joint. A similar protocol was efficiently used as an evaluative tool for the quantification of neurological disease, such as stroke, cerebral palsy, or multiple sclerosis [25,26,29,51]. The test-retest reliability resulted from good to excellent for most of the chosen indices, ranging from 0.686 to 0.969 for patients with FRDA. These results are similar to those obtained by Maurel *et al.* [39], supporting the introduction of quantitative outcome measures in clinical studies involving patients with FRDA.

The aim of this study was first to verify the sensitivity of quantitative outcome measures. All the selected robotic indices, except for two (i.e. the *Peak Velocity* and *Amplitude of Submovements*), were found to be significantly different between healthy and FRDA patients, indicating the ability of the selected outcomes to discriminate between the two groups. In particular, we also chose redundant indices to better exploit the internal coherence among indices that are related to similar feature of the motion. We found that patients with FRDA showed a significant increase of the *Number of Submovements*, and a decrease in their duration (*Duration of Submovements*) while the amplitude values (*Amplitude of Submovements*) did not show a statistically significant difference. In addition, we found a decrease in smoothness in patients with FRDA, as highlighted by the increase in the *Normalized Jerk* and the decrease in *Speed Metric.* Loss of smoothness and increase of submovements in patients with FRDA is probably related both to the compensative strategy with sudden change of acceleration, and to the decrease of the nervous system control in correctly planning the movement.

All the selected indices showed a deterioration in accuracy for patients, compared to healthy subjects. Actually, trajectories performed by the patients appear to be more circuitous, as highlighted by both the higher values of *Length Ratio* and *Lateral Deviation*. Moreover, the increase of the *Aiming Angle* showed a difficulty in the planning of the movement, moving the arm toward the direction of the target. Consequently, the kinematic indices showed a significant increase in the time required to reach the target (*Duration of Movement*), relative to healthy subjects. These results are in accordance with all the studies that analyzed quantitatively the movements in patients with FRDA, both in reaching [3,35,39] and in different tasks [39]. In fact, the slowness in task execution seems to be a key feature of ataxic movements, as was also confirmed by other authors [3,30,32-35].

It is worthy to note that in FRDA the complexity of the neurological phenotype due to the intricate interplay between the cerebellar degeneration, the somatosensory loss and the muscle atrophy does not allow a univocal interpretation of the results that we obtained. What we observed, in accordance with the previous study [52] and in correlation with the status of the disease evaluated by SARA scale, was a progressive deterioration of the movement. We could speculate that, in accordance with Corben and colleagues [52], the prolonged movement execution time in FRDA is a likely consequence of the cerebellar and spinocerebellar dysfunction. In the task execution exploited in our research, it is equally crucial to take into account the role of the visual and the motor planning impairment, widely already studied in FRDA [52-56], and not specifically addressed in the current plan.

The authors [52] suggest that cognitive impairment in people with FRDA could be related to a disruption of the cerebro-ponto-cerebello-thalamo-cerebral loops, due to the cerebellar impairment, reflecting a failure to access prefrontal/anterior regions that are necessary for an effective management of preplanning of movement and online error correction. fMRI study in healthy young-adults demonstrates the involvement of the above mentioned areas during visual observation of point-to-point reaching, using InMotion Arm Robotic device, during both real arm and virtual reaching observation [57]. This also means that healthy young adults, naturally, associate real and virtual reaching movement, despite this aspect needs to be deeper addressed in patients with FRDA.

In our experience, the number and the shape of submovements in reaching tasks in subjects with FRDA were not yet examined and the only work that analyzed smoothness in FRDA patients was conducted by Maurel *et al.* [39] showing results similar to the ones we described. It is worth noting that our set of quantitative indices better provides a measure of smoothness, which can be useful to quantify the natural progression of the disease and the eventual benefits of new therapeutic approaches. Analysis of the results has highlighted that *Duration of Movement, Normalized Jerk* and *Number of Submovements* were the best discriminative indices, as they were directly and easily measurable and correlated with the status of the disease, measured by SARA. Actually, these measures showed a strong correlation between them and a moderate correlation with the SARA total score and the upper limb score. These correlations become even stronger if we take into account only the eleven subjects older than ten years (see Table 4). As has been reported in the pilot study for age-validation of SARA [11], functional outcomes appears age-related under 10 years and are likely to be affected by poor coordination, although not necessarily pathological. In children, fine motor skills and coordination are related to the nervous system maturation and particularly to the cerebellar development that is known to be delayed in relation to the rest of the brain. Therefore, we argued that the use of the observed robotic indices in a simple reaching task for upper limb could be used both in younger ambulatory patients (older than 10 years) and in weaker adult patients with limited movements. In particular our population, although not numerous, was quite representative of the disease, with a wide range of locomotor function and disease severity and with typical onset in the first decade of life. In our opinion it is worth noting that also for patients with severe impairment, the robotic device is still capable of measuring movements and providing meaningful data. It is known that one of the drawbacks of functional scales is the ceiling effect, and also SARA showed a modest ceiling effect especially for scores greater than 30, namely for most severely affected patients. The analysis of simple indices – such as *Duration of Movement*, *Normalized Jerk* and *Number of Submovements* of reaching movement – permits: (i) an objective evaluation of motor performance of upper limb and (ii) a better exploitation of some features of ataxic movements that are not fully assessed by the SARA scale. The previously findings are relevant to accurately and efficiently quantify changes over time also when the functional scales appear no longer sensitive, suggesting their use in addition to the traditional evaluations of patients’ dexterity.

This work is, to our knowledge, the first study that quantifies the upper limb motor performance of a sample of young patients with FRDA by using a robotic device compared to a clinical functional scale.

However, the small number of our cohort and the lack of a follow up does not allow us to generalize to a larger population or to detect the sensitivity to change over time of these indices. Further analysis is needed to establish the validity of this robotic tool in a greater cohort of FRDA patients.

There are other few limitations to the current study that merit consideration. The first is due to the restriction of the In Motion Arm Robot device that allows the movement only in the horizontal plane with the involvement of the only proximal limb joints. Distal forearm and manual dexterity are not involved in the execution of the task. A further limitation is that, as mentioned before, in patients with FRDA a lot of complex neurological components as limb ataxia, sensory loss, difficult motor planning with slowed information processing, muscle weakness and not least the visual impairment could concurrently affect the simple virtual reaching task selected for this study. Finally, the result of this study were based on the only dominant limb. In a future longitudinal extension of this work, we will analyze also the non dominant limb, as suggested by Corben *et al.* [3]. Furthermore, future study should also address the influence of specific sensory-motor integration on this task execution.

## Conclusions

Overall, this study shows that the use of robotic indices may be used as a reliable and sensitive clinical measurement tool for assessing upper limb motor function in the population with FRDA. Further, the outcome measures obtained by means of robotic devices can improve the sensitivity of clinical evaluations of patients’ dexterity, supporting the clinical decision-making, and can accurately and efficiently quantify changes over time in clinical trials, particularly when functional scales appear to be no longer sensitive as in the case of patients with severe functional impairment.

## Abbreviation

FRDA: Friedreich’s ataxia
SARA: Scale for the Assessment and Rating of Ataxia
GAA: Guanine–Adenine–Adenine
FARS: Friedreich Ataxia Rating Scale
ICARS: International Cooperative Ataxia Rating Scale
BARS: Brief Ataxia Rating Scale
SCARA: Selective Compliance Assembly Robot Arm
D: Duration of movement
MV: Mean velocity
PV: Peak velocity
LR: Length ratio
LD: Lateral deviation
AA: Aiming angle
NJ: Normalized jerk
SM: Speed metric
NS: Number of submovements
DS: Duration of submovements
AS: Amplitude of submovements
ICC: Intraclass correlation coefficient
